# How many days of monitoring predict physical activity and sedentary behaviour in older adults?

**DOI:** 10.1186/1479-5868-8-62

**Published:** 2011-06-16

**Authors:** Teresa L Hart, Ann M Swartz, Susan E Cashin, Scott J Strath

**Affiliations:** 1 Department of Health Sciences, Arizona State University, Phoenix, Arizona, USA; 2 Department of Human Movement Sciences, University of Wisconsin-Milwaukee, Milwaukee, Wisconsin, USA

## Abstract

**Background:**

The number of days of pedometer or accelerometer data needed to reliably assess physical activity (PA) is important for research that examines the relationship with health. While this important research has been completed in young to middle-aged adults, data is lacking in older adults. Further, data determining the number of days of self-reports PA data is also void. The purpose of this study was to examine the number of days needed to predict habitual PA and sedentary behaviour across pedometer, accelerometer, and physical activity log (PA log) data in older adults.

**Methods:**

Participants (52 older men and women; age = 69.3 ± 7.4 years, range= 55-86 years) wore a Yamax Digiwalker SW-200 pedometer and an ActiGraph 7164 accelerometer while completing a PA log for 21 consecutive days. Mean differences each instrument and intensity between days of the week were examined using separate repeated measures analysis of variance for with pairwise comparisons. Spearman-Brown Prophecy Formulae based on Intraclass Correlations of .80, .85, .90 and .95 were used to predict the number of days of accelerometer or pedometer wear or PA log daily records needed to represent total PA, light PA, moderate-to-vigorous PA, and sedentary behaviour.

**Results:**

Results of this study showed that three days of accelerometer data, four days of pedometer data, or four days of completing PA logs are needed to accurately predict PA levels in older adults. When examining time spent in specific intensities of PA, fewer days of data are needed for accurate prediction of time spent in that activity for ActiGraph but more for the PA log. To accurately predict average daily time spent in sedentary behaviour, five days of ActiGraph data are needed.

**Conclusions:**

The number days of objective (pedometer and ActiGraph) and subjective (PA log) data needed to accurately estimate daily PA in older adults was relatively consistent. Despite no statistical differences between days for total PA by the pedometer and ActiGraph, the magnitude of differences between days suggests that day of the week cannot be completely ignored in the design and analysis of PA studies that involve < 7-day monitoring protocols for these instruments. More days of accelerometer data were needed to determine typical sedentary behaviour than PA level in this population of older adults.

## Background

Physical activity (PA) is a sporadic and complex behaviour to measure and is subject to inter- and intra-individual variability [1]. It has also been suggested that sedentary behaviour is an important, independent behaviour to account for due to its relation with health [2]. Self-report methods to assess PA and sedentary behaviour such as logs, questionnaires, or surveys, are often easy to administer however are subject to error and recall bias [3]. Objective measures of PA and sedentary behaviour, such as pedometers and accelerometers, have shown promise when used to assess habitual behaviour. Determining the number of days to reliably assess habitual PA and sedentary behaviour and minimizing participant burden remains a challenge.

The number of days to reliably predict habitual PA behaviour for young and middle-aged adults has been examined using pedometers and accelerometers. As part of a year-long pedometer self-monitoring study in adults (mean age = 38 ± 10 years), it was determined that five consecutive days or six randomly selected days of data were needed to produce an intraclass correlation (ICC) of 0.80 [4]. Data from seven days of consecutive pedometer monitoring in adult men (mean age = 49.1 ± 16.2 years) and women ( mean age = 44.8 ± 16.9 years) suggested that three days of monitoring produced an ICC of 0.80 or greater [5]. Similar results were reported when assessing PA and sedentary behaviour using an accelerometer in middle-aged adults. To reliably predict 21 days of monitoring, it was suggested that three to four days of accelerometer monitoring were needed to achieve 80% reliability for total PA as well as moderate and vigorous intensity PA, and seven days of monitoring were needed to predict sedentary behaviour [6]. Together, these data suggest that a minimum of three days of objective monitoring are needed to reliably predict PA behaviour, while seven days of objective monitoring are needed to predict time spent in sedentary behaviour in a young to middle-aged population.

Despite the popularity of estimating PA and sedentary behaviours to determine PA or SB prevalence or relationships with various aspects of health, there remain a number of gaps in the literature focusing on the number of data acquisition days needed to reliably predict PA and sedentary behaviours. First, while studies have reported consistent results with regards to number of days of monitoring using objective methods of PA assessment in a young to middle-aged population, data on older adults is lacking. Second, the number of days of data acquisition needed to reliably predict PA behaviour from subjective methods is void in the literature. Finally, comprehensive, concurrent comparisons across subjective and objective methods of PA and sedentary behaviour assessment within the same population are limited. Therefore the purpose of this study was to examine the number of data acquisition days needed to reliably predict PA and sedentary behaviour using a pedometer, accelerometer, or PA log in an older adult population. Secondly, we aim to provide an indication of whether estimates of PA and sedentary behaviour vary depending on which days of the week are examined.. The results from this study will provide useful information regarding the use of PA assessment methodology for the older adult population.

## Methods

### Participants and Procedures

Participants included 52 older men and women (mean ± standard deviation age = 69.3 ± 7.4 years, range = 55-86 years) who were recruited as part of a larger ongoing trial examining objectively determined physical activity profiles of community dwelling older adults. Participants wore a Yamax Digiwalker SW-200 (Yamasa Corporation, Tokyo, Japan) pedometer and an ActiGraph model 7164 (formerly CSI and MTI; ActiGraph LLC, Pensacola, FL) accelerometer concurrently while completing a physical activity log (PA log) during all waking hours (excluding showering and swimming) for 21 consecutive days. Table 1 contains demographic information for all participants. Ethical approval for this study was granted by the University of Wisconsin-Milwaukee Institutional Review Board.

**Table 1:** Participant demographics and physical activity behaviour data.

	All (N = 52)	Male (n = 13)	Female (n = 39)
**Age (years)**	69.3 (7.4)	68.7 (10.2)	69.6 (6.3)
			
**Body Mass Index (kg/m**^**2**^**)**	27.0 (23.9, 29.3)	27.0 (24.6, 30.2)	27.0 (23.6, 29.4)
			
**Waist Circumference (cm)**	94.5 (83.9, 101.0)	100.0 (92.0, 111.0)	90.3 (81.6, 100.4)
			
**Resting Systolic Blood Pressure (mmHg)**	128 (14)	127 (13)	128 (14)
			
**Resting Diastolic Blood Pressure (mmHg)**	77 (9)	74 (9)	78 (8)
			
**Accelerometer (counts/day)**	250550 (116260)	278430 (152809)	241257 (102040)
			
**PA Log (MET-min/day)**	1107 (358)	1147 (496)	1094 (307)
			
**Pedometer (steps/day)**	5589 (3941, 8971)	8971 (3894, 9260)	5490 (3892, 7592)

Note. Data are presented as mean (standard deviation) or median (interquartile range).

### Instruments

#### ActiGraph Accelerometer

The ActiGraph (model 7164) used in this study is one of the most widely used accelerometers in PA research. This same model has been used for objective PA monitoring in the National Health and Nutrition Examination Survey (NHANES) [7]. Detailed technical specification for the ActiGraph are provided elsewhere [8,9]. The primary outputs from the ActiGraph are activity counts, which represent raw accelerations that have been filtered, digitized, integrated and rescaled. Detected activity counts are summed over each epoch (i.e., commonly a minute in length for adults) [10]. The sum of the activity counts in a given epoch is related to activity intensity and can be categorized (e.g., sedentary, light, moderate, vigorous) based on validated activity count cutpoints [8,9]. For the current study, the ActiGraph was worn on the right side of the waist on an elastic belt, according to manufacturer specifications.

#### Yamax Digiwalker SW-200 Pedometer

The Yamax Digiwalker SW-200 is an electronic pedometer with a horizontal, spring-suspended lever arm which moves up and down with vertical accelerations of the hip. When accelerations are ≥ 0.35 g, the lever arm makes an electrical contact and one step is recorded. The Yamax displays the number of steps taken during a given period of time during which the monitor is worn with a display output range of 0-99,999 steps. The Yamax attaches to the waist line of pants or to a belt at the midline of either thigh. The Yamax Digiwalker is considered to be the criterion pedometer for free-living PA research studies [11].

#### Physical Activity Log

Participants recorded activities on the PA log which has been used by others in past studies [12] at the end of each day. A list of activities was provided for the participant on the daily log sheet and the broad categories included: household activities, lawn/garden activities, volunteer/occupational, care of others, transportation, walking, dancing, sports, conditioning, and inactivity. Along with providing information regarding the type of activity performed, participants were also asked to record how long each activity was performed. MET values were assigned to each activity [13] and multiplied by the number of minutes each activity performed resulting in MET-min. All PA logs were checked by a researcher and reviewed with each participant to ensure completeness. Because the PA log did not have postural determination components, we combined sedentary behaviours with light intensity PA into one intensity category. Total MET-min/day were calculated for each day in addition to MET-min/day in each of the two intensity categories (i.e., sed/light and moderate-to-vigorous).

### Data Treatment and Statistical Analysis

To determine total PA performed each day, total activity counts from the ActiGraph, total number of steps/day from the Yamax, and total MET-min/day from the PA log were utilized in analyses. ActiGraph data were screened for non-wear time using 60 consecutive zero counts/minute. ActiGraph cutpoints of 0-50 counts/min were considered to be sedentary behaviour (based on cutpoints suggested by Esliger et al. [14] and Crouter et al. [15] and found to be representative of sitting/lying behaviour [16]), 51-759 counts/min were considered to be light PA [17], 760-1951 counts/min were considered to be moderate lifestyle PA, and ≥1952 counts/min were considered to be moderate- to vigorous-intensity PA [8]. For the PA log, activities with MET values ranging from 1-2.99 were considered sed/light, ≥3 were considered moderate- to vigorous-intensity PA [18]. The resulting data for the PA log was total MET-min/day, and MET-min/day in each intensity category. Non-normally distributed data were transformed using log + 1 for inferential analyses. Descriptive statistics were presented in their raw form (i.e., not transformed).

To address the main purpose of this paper, Spearman-Brown Prophecy Formulas based on ICC for all 21 days and a reliability of .80 were used to predict the number of days of complete data needed to represent total PA (Yamax steps, total ActiGraph counts, and total PA log MET-min/day), sedentary behaviour (total minutes from accelerometer with ≤ 50 activity counts/min), light-intensity(ActiGraph min/day and PA log MET-min/day), moderate-intensity (ActiGraph min/day), vigorous-intensity (ActiGraph min/day), and moderate-to vigorous-intensity (ActiGraph min/day and PA log MET-min/day) PA. These analyses were repeated to calculate a reliability of 0.85, 0.9, and 0.95.

To address the secondary aim of this paper, a repeated measures analysis of variance (RMANOVA) with *post hoc *pairwise comparisons where necessary was used to determine between day differences in mean PA level (i.e., the seven days of the week) for each intensity and for all instruments. To derive daily mean PA estimates from the 21 days, data from each day of the week was averaged (e.g., the mean of the three Mondays included in the monitoring period were averaged to determine the average PA behaviour on Monday). Analyses were completed using SAS version 9.1 (Cary, IL) and SPSS version 17 (Chicago, IL). Statistical significance for all analyses was set at p < .05.

## Results

The mean wear/completion time was 823.1 ± 103.3 minutes/day. Total PA from the ActiGraph, Yamax and PA log were all non-normally distributed and therefore log transformed for inferential analysis. All log transformations resulted in normal distributions.

### Number of Days of Complete Data Needed to Predict PA Behaviour

The number of days of complete data needed to predict 21 days of total PA behaviour in this older adult population was consistent, ranging from three days (ActiGraph) to four days (Yamax and PA log; Table 2). More days of complete data (five days) were needed to reliably predict sedentary behaviour from the ActiGraph compared the number of days needed to predict time spent in light- (ActiGraph: 3 days), moderate- (ActiGraph: 2 days), and vigorous-intensity PA (ActiGraph: 2 days). Further, these results show the self-report of PA behaviour requires more days of complete data (14 days for light-intensity PA and 5 for moderate- to vigorous-intensity PA), compared with the ActiGraph (3 days for light intensity PA and 4 days for moderate- to vigorous-intensity PA), to reliably predict 21 days of light-intensity and moderate- to vigorous- intensity PA behaviour (Table 2).

**Table 2:** Number of complete data acquisition days needed to predict 21 days of physical activity behavior from objective and subjective methodologies as determined by Spearman Brown Prophecy Formula.

	ActiGraph 7164				Yamax SW-200				Physical activity log			
	
Reliability Value	0.80	0.85	0.90	0.95	0.80	0.85	0.90	0.95	0.80	0.85	0.90	0.95
**Total PA**	3	4	6	13	4	6	10	21	4	5	8	18
**Sedentary Behavior**	5	7	11	21	--	--	--	--	--	--	--	--
**Light-Intensity PA**	3	4	7	14	--	--	--	--	14	20	21	21
**Moderate-Intensity Lifestyle PA**	2	3	5	10	--	--	--	--	--	--	--	--
**MVPA**	2	3	5	10	--	--	--	--	5	7	12	21

Note. Physical activity (PA); moderate-to vigorous-intensity physical activity (MVPA)

Note. ActiGraph cutpoints used for this analysis were 0-50 counts/min = sedentary behavior; 51-759 counts/min = light intensity physical activity; 760-1951 = moderate lifestyle physical activity; ≥1952 = moderate-to-vigorous physical activity (Crouter et al., 2006; Matthews, 2005; Freedson et al., 1998). For the PA log, Light PA = activities ranging from 1-2.99 METs and MVPA = activities ≥3 METs (Pate et al., 1995).

Note. Physical activity log light intensity physical activity is inclusive of sedentary behavior

### Which days of the week can be used to predict total PA behaviour?

Total PA from the ActiGraph, Yamax and PA log by day of the week are presented in Table 3 as the mean value for that day of the week (95% confidence interval; 95% CI) based on the 21 days of monitoring performed (e.g., data for Monday represents the average of the three Mondays that were monitored during the 21 monitoring period). Results from the individual RMANOVA for the ActiGraph and for the Yamax showed no significant differences in total PA level between days of the week (p = .17 for both the ActiGraph and the Yamax). There was a significant difference in total PA level observed between days of the week by the PA log (p = .04). Results of the *post hoc *pairwise comparisons are displayed on Table 3.

**Table 3:** Average total daily physical activity level as assessed by ActiGraph, Yamax, and Physical Activity Log.

	Sunday	Monday	Tuesday	Wednesday	Thursday	Friday	Saturday
**ActiGraph Total Activity Counts/day**	211205 (150350, 325184)	236379 (171532, 344185)	218298 (155461, 316880)	233436 (153418, 318170)	229008 (142342, 346925)	223605 (138209, 303186)	230597 (139701, 297831)
**Yamax Total Steps/day**	5790 (3412, 8836)	6186 (4037, 9249)	6022 (3403, 8663)	6783 (3618, 8850)	6030 (3921, 9167)	5150 (3692, 8330)	5431 (3701, 7795)
**PA Log Total MET-min/day**	980 (713, 1353)^g^	971 (803, 1284)	992 (768, 1287)^g^	1036 (841, 1322)^c^	972 (754, 1281)^b,g^	1050 (896, 1300)^a^	1112 (918, 1331)^a,c^

Note. Data presented represent the average value for each day of the week calculated from original 21 days of data. Data are presented as median (interquartile range).

Note. Superscript 'a' represents days which are significantly different from Sunday; 'b' represents different from Monday; 'c' represents different from Tuesday; 'd' represents different from Wednesday; 'e' represents different from Thursday; 'f' represents different from Friday; and 'g' represents different from Saturday. Significant differences determined using Repeated Measures Analysis of Variance (log transformation was used on data for all days for analysis where data were non-normally distributed). p < .05 was used for statistical significance.

Data for ActiGraph variables light intensity PA and sedentary behaviour were normally distributed; all other ActiGraph variables were non-normal and log transformed for inferential analysis. Table 4 shows the means (95% CI) for ActiGraph-determined sedentary behaviour, light PA, moderate lifestyle PA, and moderate- to vigorous-intensity PA for each day of the week. Data for all PA log variables were non-normally distributed and log transformed for inferential analysis. The means (95% CI) for PA log-estimated MET-min/day in sed/light, moderate- to vigorous-intensity PA are also presented in Table 4.

**Table 4:** Daily estimated time spent in physical activity intensities by ActiGraph (minutes/day) and physical activity log (MET-min/day).

	Sunday	Monday	Tuesday	Wednesday	Thursday	Friday	Saturday
Sedentary Behavior* (min/day)^2^	424.7 (392.7, 456.7)	412.3 (382.7, 441.9)	421.1 (389.1, 453.1)	437.1 (406.4, 467.7)	427.8 (395.9, 459.7)	423.1 (390.6, 455.6)	412.9 (381.6, 444.3)
Light PA* (min/day)^2^	309.3 (285.4, 333.1)	313.5 (291.1, 335.8)	312.6 (293.6, 331.6)	312.3 (291.2, 333.4)	314.5 (292.0, 337.9)	313.4 (291.2, 335.6)	324.1 (301.0, 347.1)
**Sed/Light PA**^† ^**(**MET-min/day)^3^	713.2 (493.1, 971.3)	781.1 (596.6, 983.8)^a^	710.4 (556.3, 942.5)^a^	682.1 (559.7, 1075.6)^a^	692.5 (569.7, 925.3)	749.2 (550.8, 1008.8)^a^	735.0 (568.2, 1083.3)^a^
**Moderate Lifestyle PA**^† ^(min/day)^2^	64.5 (35.6, 92.6)^c,d,e,f^	75.3 (45.3, 110.9)^c,e,f,g^	68.3 (48.1, 98.7)^a^	60.0 (40.2, 104.7)^b^	79.9 (64.9, 95.0)^a,b^	67.0 (36.4, 108.5)^a,c^	69.25 (37.1, 113.4)^c^
**MVPA**^† ^(min/day)^2^	12.3 (4.5, 25.3)	15.7 (6.5, 39.0)	13.7 (6.5, 25.7)	15.0 (7.7, 31.5)	12.0 (5.2, 30.0)	13.0 (6.8, 29.8)	14.7 (7.5, 27.8)
**MVPA**^† ^**(**MET-min/day) ^3^	143.5 (57.8, 363.9)	249.1 (78.1, 474.4)	227.0 (92.5, 404.3)	223.0 (77.6, 329.7)	282.0 (159.5, 515.8)	278.0 (112.8, 478.0)	270.9 (97.5, 455.0)

Note. Data presented represent the average value for each day of the week calculated from original 21 days of data. Data are presented as *mean (95% confidence interval) or ^†^median (interquartile range).

Note. ^2^, ActiGraph data; ^3^, Physical Activity Log data

Note. Physical activity (PA); moderate-to-vigorous physical activity (MVPA). ActiGraph cutpoints used for this analysis were 0-50 counts/min = sedentary behavior; 51-759 counts/min = light intensity physical activity; 760-1951 = moderate lifestyle physical activity; ≥1952 = moderate-to-vigorous physical activity (Crouter et al.[15]; Matthews [17]; Freedson et al.[8]).

Note. For the PA log, sedentary behavior/light intensity physical activity (Sed/Light PA); moderate-to vigorous intensity physical activity (MVPA). Sed/Light PA = activities ranging from 1-2.99 METs and MVPA = activities ≥3 METs. (Pate et al.[18])

Note. Superscript 'a' represents days which are significantly different from Sunday; 'b' represents different from Monday; 'c' represents different from Tuesday; 'd' represents different from Wednesday; 'e' represents different from Thursday; 'f' represents different from Friday; and 'g' represents different from Saturday. Significant differences determined using Repeated Measures Analysis of Variance (^†^log transformation was used on data for all days for analysis where data were non-normally distributed). p < .05 was used for statistical significance.

Results of the RMANOVA showed no significant differences between days of the week for sedentary behaviour and light PA based on ActiGraph data (p = .48 and .58, respectively; Table 4). However, when examining the data from the PA log, results showed significant differences between days of the week for MET-min/day in sed/light PA (p = .004). *Post hoc *pairwise comparisons showed all days were significantly different from Sunday except for Thursday (Table 4).

Significant differences between days of the week were present when estimating moderate-intensity lifestyle PA (p = .01) and moderate-to vigorous-intensity PA (p = .011) using data from the ActiGraph. No consistent patterns of difference between days can be identified from the *post hoc *pairwise comparisons (Table 4). Examination of PA level by log data showed no significant difference between days of the week for MET-min/day in moderate- to vigorous-intensity PA (p = .06; Table 4).

## Discussion

This study examined the number of complete data acquisition days needed to reliably estimate 21 days of PA and sedentary behaviour using objective instruments (ActiGraph accelerometer and Yamax Digiwalker pedometer) and a subjective instrument (PA log) in an older adult population. Results of this study suggest three to four days of complete data are required by all instruments to reliably predict total PA behaviour. When the outcome variable of interest is time spent in a specific intensity of physical activity, the number of days needed to reliably predict PA behaviour is dependent on the instrument used. Two to three days of data acquisition from the ActiGraph will provide reliable estimates of light-, moderate lifestyle-, and moderate- to vigorous-intensity PA, whereas five to 14 days (depending on the intensity) of complete data acquisition are needed to predict intensity specific PA behaviour from a PA log. Sedentary behaviour, captured in this study only by the ActiGraph, required 5 days of complete data to reliably predict total time spent in sedentary behaviour.

The results of this study are similar to previously published findings using objective assessment tools. A study of 365 days of pedometer data acquisition in adults (mean age = 38.0 ± 9.9 years) concluded that five consecutive days or six random days were needed to reliably estimate habitual PA, however no specific days of the week were recommended [4]. Tudor-Locke et al. [5] collected seven days of pedometer data in adults (mean age = 49.1 ± 16.2 years) and suggested that three days of pedometer monitoring were sufficient to reliably estimate habitual PA. Further, significant differences in PA level between days of the week were reported by Tudor-Locke et al [5] with the primary difference being Sunday. In spite of these daily differences, the authors suggested any three days of monitoring would be sufficient [5]. Rowe and colleagues [19] examined data from older adults (mean age = 74.0 ± 9.5 years) who wore a pedometer for 7 days. Results showed no differences in steps/day between days of the week, with the exception of Sunday and authors concluded that two days of data collection were needed to reliably predict steps/day [19]. Our results add to this current literature set by suggesting that a minimum of four days of complete pedometer data are needed to reliably estimate total PA level in an older adult population. However, despite the lack of statistical difference in steps/day between days, it should be noted that there is a clinically significant difference in the number of steps taken per day when examined by day of the week. For instance, the average daily steps taken during the 21 day monitoring period on Friday were 5312, while 6601 steps were taken on Sunday. This difference equates to almost 1300 steps per day, or over 1/2 mile [20-22]. When looking at the efficacy of a pedometer intervention, an increase of 1000 steps per day may be clinically significant, based on the baseline steps taken. Therefore, the authors recommend that the impact of day of the week be considered in designing and analyzing PA studies, for example in planning for appropriate coverage of each day of the week in the overall group if a < 7-day protocol is used, and to control for possible day of the week effects by matching or statistical adjustment when repeated assessments are used (e.g. in intervention studies).

When predicting intensity specific PA behaviour using objective monitoring, it is apparent that the number of days of complete data needed to reliably predict behaviour are less as the intensity of PA increases. Moderate- to vigorous-intensity PA is generally planned, predictable, and less variable in older adults, thereby requiring fewer days of complete data to reliably predict the behaviour [19]. However, sedentary behaviour and light-intensity PA are less predictable on a day-to-day basis, and therefore require more monitoring days to reliably predict the behaviour. Similar to the results of the current study that suggest two- to three-days of complete data acquisition are needed to reliably predict PA level, Rowe and colleagues [19] concluded that two days of data collection were needed to reliably predict seven-days of moderate- to vigorous-intensity PA in an older adult population [18]. Further, Matthews et al. [6] reported three to four days of monitoring were needed to predict 21 days of moderate-intensity lifestyle (i.e., 500-1951 counts/min), and moderate- to vigorous-intensity (i.e., ≥ 1952 counts/min) PA in middle-aged men and women [6]. When examining sedentary behaviour, the number of days needed to reliably predict behaviour increases. Matthews et al. [6] suggested seven days of monitoring to reliably assess inactivity (i.e., 0-499 activity counts/minute) compared with five days of complete data needed to reliably predict sedentary behaviour (i.e., 0-50 activity counts/minute) in the current study. Together these data suggest that more days of data acquisition are needed to reliably estimate sedentary behaviour compared with PA behaviour of at least a moderate intensity.

Results from this study and published literature suggest that fewer days of monitoring are needed when objective PA assessment tools are used, compared with subjective PA assessment tools. Results showed little day-to-day differences between days of the week as assessed by both a pedometer and accelerometer, but larger variances were seen in the PA log data, especially when examining sedentary and light intensity behaviours (up to a 146 MET min/day difference between days). Previous research has demonstrated that individuals are better able to recall PA of moderate- to vigorous-intensity, compared with those of lesser intensities [23], due to the ubiquitous nature of sedentary and light intensity activities, and the fact that many moderate- and vigorous-intensity activities are generally planned and purposeful [24]. Therefore, results of this study support previous published conclusions suggesting more days of monitoring are needed to reliably predict PA from self-report instruments. Our data adds to the current literature by making concurrent assessments of the number of days of data acquisition needed to reliably predict PA behaviour by subjective and objective PA methodologies.

Seven-day monitoring protocols, which are often employed in studies that examine PA as either an outcome variable, or as an independent variable, would achieve reliability of ICCs approximately 0.85 to 0.90 for all measures of PA and sedentary by the accelerometer; approximately 0.85 for the pedometer and approximately 0.85-0.90 for total or MVPA by the PA log, given 100% compliance. A 7-day monitoring period could reliably predict light intensity activity by the accelerometer but not by the log. Additionally, the seven day monitoring period avoids issues related to day-to-day variation of PA behaviour. Our data support the continued use of a 7-day PA monitoring period to reliably predict PA and sedentary behaviour.

This study provides useful information regarding the utilization of multiple methods of PA assessment in the field, however it has some limitations. First, this population was fairly homogeneous in age and health status which may cause a threat to external validity. Additionally, there were relatively few males compared to females which may have resulted from volunteer bias. However, this type of homogeneous sample was also seen in related literature [4,5,19]. Also, there was only a mean of 1.3 minutes of vigorous intensity PA as detected by the accelerometer which resulted in the combination of moderate and vigorous intensity PA for the analysis. This practice is consistent with Troiano et al. [7]. The data reported in this paper focused on reliability when using some common cutpoints (which were not designed specifically for an older population), however given the similarity in the required number of days of monitoring for moderate-lifestyle (760 - 1951 counts/min) and moderate-to-vigorous (1952+ counts/min) PA would suggest that these results are not unduly affected by choice of cutpoints. Similarly, the low amount of MET-min/day seen in vigorous intensity PA by the PA log resulted in the combining of the moderate and vigorous intensity PA categories. Finally, there was no power calculation for this study. However, the sample size of the current study is in the mid-range of samples in related literature [4,5,19].

## Conclusions

This study concurrently examined the number of days of monitoring to reliably estimate habitual PA and sedentary behaviour from objective and subjective PA assessment methods. Based on the results of this study, 3-4 days of monitoring are needed to assess habitual PA regardless of which instrument is selected. Despite no statistical differences between days for total PA by the pedometer and ActiGraph, the magnitude of differences between days suggests that day of the week cannot be completely ignored in the design and analysis of PA studies that involve < 7-day monitoring protocols for these instruments. If specific intensities of PA are an outcome of interest, additional days may be needed for the PA log but not the accelerometer. For sedentary behaviour, any 5 days of monitoring will provide a reliable estimate of behaviour.

## Competing interests

The authors declare that they have no competing interests.

## Authors' contributions

TH was involved with data analysis and manuscript preparation. SS and AS were involved with data collection and manuscript preparation. SC was involved with data analysis. All authors read and approved the final manuscript.
